# The burden of cardiovascular morbidity in a European Mediterranean population with multimorbidity: a cross-sectional study

**DOI:** 10.1186/s12875-016-0546-4

**Published:** 2016-11-03

**Authors:** Concepción Violán, Néker Bejarano-Rivera, Quintí Foguet-Boreu, Albert Roso Llorach, Mariona Pons-Vigués, Miguel Martin Mateo, Enriqueta Pujol-Ribera

**Affiliations:** 1 Institut Universitari d’Investigació en Atenció Primària Jordi Gol (IDIAP Jordi Gol), Gran Via Corts Catalanes, 587 àtic, 08007 Barcelona, Spain; 2 Department of Paediatrics, Obstetrics and Gynaecology and Preventative Medicine. Facultad de Medicina, Universitat Autònoma de Barcelona, Edifici M. Campus Universitari UAB, 08193 Bellaterra (Cerdanyola del Vallès), Spain; 3 Universitat Autònoma de Barcelona, Bellaterra (Cerdanyola del Vallès), Barcelona, Spain; 4 Emergency Department, University Hospital of Vic, Francesc Pla el Vigatá, 08500 Vic Barcelona, Spain; 5 Department of Medical Sciences, School of Medicine, University of Girona, Emili Grahit, 77, 17071 Girona, Spain; 6 Faculty of Nursing, University of Girona, Emili Grahit, 77, 17071 Girona, Spain

**Keywords:** Multimorbidity, Cardiovascular morbidity, Cardiovascular risk factors, Comorbidity, Electronic health records, Primary health care

## Abstract

**Background:**

Cardiovascular diseases are highly represented in multimorbidity patterns. Nevertheless, few studies have analysed the burden of these diseases in the population with multimorbidity. The objective of this study was to identify and describe the cardiovascular diseases among the patients with multimorbidity.

**Methods:**

We designed a cross-sectional study in patients ≥19 years old assigned to 251 primary health care centres in Catalonia, Spain. The main outcome was cardiovascular morbidity burden, defined as the presence of one or more of 24 chronic cardiovascular diseases in multimorbid patients (≥2 chronic conditions). Two groups were defined, with and without multimorbidity; the multimorbidity group was further divided into cardiovascular and non-cardiovascular subgroups. The secondary outcomes were: modifiable major cardiovascular risk factors (smoking, hypertension, hypercholesterolaemia, diabetes) and cardiovascular risk score (REGICOR, *Registre Gironí del Cor*). Other variables analysed were: sex, age (19–24, 25–44, 45–64, 65–79, and 80+ years), number of chronic diseases, urban setting, active toxic habits (smoking and alcohol), physical parameters and laboratory tests.

**Results:**

A total of 1,749,710 individuals were included (mean age, 47.4 years [SD: 17.8]; 50.7 % women), of which nearly half (46.8 %) had multimorbidity (95 % CI: 46.9–47.1). In patients with multimorbidity,, the cardiovascular burden was 54.1 % of morbidity (95 % CI: 54.0–54.2) and the four most prevalent cardiovascular diseases were uncomplicated hypertension (75.3 %), varicose veins of leg (20.6 %), “other” heart disease (10.5 %) and atrial fibrillation/flutter (6.7 %). In the cardiovascular morbidity subgroup, 38.2 % had more than one cardiovascular disease. The most prevalent duet and triplet combinations were uncomplicated hypertension & lipid disorder (38.8 %) and uncomplicated hypertension & lipid disorder & non-insulin dependent diabetes (11.3 %), respectively. By age groups, the same duet was the most prevalent in patients aged 45–80 years and in men aged 25–44 years. In women aged 19–44, varicose veins of leg & anxiety disorder/anxiety was the most prevalent; in men aged 19–24, it was uncomplicated hypertension & obesity. Patients with multimorbidity showed a higher cardiovascular risk profile than the non-multimorbidity group.

**Conclusions:**

More than 50 % percent of patients with multimorbidity had cardiovascular diseases, the most frequent being hypertension. The presence of cardiovascular risk factors and the cardiovascular risk profile were higher in the multimorbidity group than the non-multimorbidity group. Hypertension, diabetes and dyslipidaemia constituted the most prevalent multimorbidity pattern.

## Background

Multimorbidity (MM), understood as the diagnosis of two or more disease diagnoses in the same patient, will become a major public challenge in the coming decades. A systematic review found that the prevalence of MM varied depending on age, and ranged from 13 % in participants aged 18 years and older to 95 % in a population aged 65 years and older [1]. The forecasts for 2050 indicate that the world population aged 60 years and older is expected to total more than 2 billion in 2050 [2]; MM is likely to increase substantially, with the consequent impact on health services [3, 4]. At the individual level, MM represents reduced physical and mental function, decreased quality of life, low quality of care, increased use of health services, major complications with treatments, and increased mortality [5]. In addition, MM is common in primary care and treatment management is a major burden for health care professionals [6].

Cardiovascular diseases represent 46 % of non-communicable disease deaths worldwide [7], and are highly represented in MM. Several recent studies have highlighted hypertension, diabetes, obesity and coronary heart disease as the most frequent MM diagnoses [1, 8, 9]. Furthermore, the concurrence of multiple cardiovascular diseases is an independent predictor of prognosis in patients with established cardiovascular disease [10]. Knowing the cardiovascular disease burden in patients with MM and how these diseases are associated could help clinicians to more effectively search for other diseases when one of them is first diagnosed. It may also contribute to the design of clinical practice guidelines and the development and management of health programs.

The purpose of this study was to describe the burden of cardiovascular diseases in patients with MM by sex and life-stage in a large population sample, evaluate the cardiovascular risk factors (CVRF) and cardiovascular risk (CVR) present a group of patients with MM, and compare these factors with a non-MM group.

## Methods

### Data source and study population

A cross-sectional study of adult residents was conducted in Catalonia, a Mediterranean region of southern Europe with 7,434,632 inhabitants (16 % of the Spanish population, 2010 census). In Catalonia, 358 primary health care teams (PHCTs) comprised of general practitioners (GPs), nurses, social workers and support personnel are assigned by geographical area and responsible for the health care of the population in their areas. The Catalan Health Institute manages 274 PHCTs (76.5 %), serving a population of 4,859,725 adults; the remaining PHCTs are managed by other providers. Primary care professionals systematically use electronic health records (EHR) to record diagnoses, prescriptions and other clinical information, patient management, and administrative activities. The Catalan Health Institute Information System for the Development of Research in Primary Care (SIDIAP) [11] compiles coded clinical information from the EHR system based on data from its 274 PHCTs. A subset of SIDIAP records meeting the highest quality criteria for clinical data (SIDIAP-Q) includes 40 % of the SIDIAP population (1,936,443 patients), attended by the 1,319 GPs assigned to 251 PHCTs whose data recording scored highest in a validated comparison process [12]. SIDIAP has been shown to be highly representative of the general Catalan population in terms of geography, age and sex distribution, according to the official 2010 census [12].

The study sample, was selected from the SIDIAP-Q database, included 1,749,710 patients aged 19 years or older, assigned to 251 PHCTs during the period of study (1 January- 31 December 2010); 186,733 individuals were excluded because they were younger than 19 years.

### Coding of diseases

International Classification of Diseases (ICD-10) codes were mapped to the International Classification of Primary Care (ICPC-2e-v.4.2, available at: http://www.kith.no/templates/kith_WebPage____1111.aspx). R codes (symptoms, signs and abnormal clinical and laboratory findings, not elsewhere classified) and Z codes (factors influencing health status and contact with health services) were excluded, resulting in 686 included codes. Each diagnosis was then classified using O’Halloran criteria for chronic disease [13]: (i) have a duration that has lasted, or is expected to last, at least 6 months; (ii) have a pattern of recurrence or deterioration; (iii) have a poor prognosis and (iv) produce consequences, or sequel, that have an impact on the individual’s quality of life [13, 14]. All results were described by ICPC-2 codes and 146 chronic diseases were included in the analysis.

Cardiovascular morbidity was studied in 24 selected cardiovascular chronic diseases from chapter K of ICPC- 2, based on active diagnoses recorded in EHR as of December 31, 2010: Rheumatic fever/heart disease (K71); Neoplasm, cardiovascular (K72); Congenital anomaly, cardiovascular (K73); Ischaemic heart disease with angina (K74); Acute myocardial infarction (K75); Ischaemic heart disease without angina (K76); Heart failure (K77); Atrial fibrillation/flutter (K78); Paroxysmal tachycardia (K79); Cardiac arrhythmia NOS (K80); Heart/arterial murmur NOS (K81); Pulmonary heart disease (K82); Heart valve disease NOS (K83); Heart disease, other (K84); Hypertension, uncomplicated (K86); Hypertension, complicated (K87); Postural hypotension (K88); Transient cerebral ischaemia (K89); Stroke/cerebrovascular accident (K90); Cerebrovascular disease (K91); Atherosclerosis/peripheral vascular disease (K92); Pulmonary embolism (K93); Phlebitis/thrombophlebitis (K94) and Varicose veins of leg (K95).

### Outcomes and variables

The main outcome was cardiovascular morbidity burden defined as the coexistence of one or more chronic cardiovascular diseases in patients with MM. MM was defined as the coexistence of 2 or more chronic diseases.

Two study groups were defined, with and without MM. The MM group was further divided into subgroups, constituting a cardiovascular morbidity group (MM-CMG) –i.e., MM patients with one or more chronic cardiovascular disease– and non-cardiovascular morbidity group with other chronic diseases (MM-NCMG).

Secondary outcomes included CVR and CVRF profile. CVR was assessed by the REGICOR (Registre Gironí del Cor) score, which evaluates the 10-year risk of a coronary event (angina, myocardial infarct with/without symptoms, fatal or non-fatal), with four categories of severity: low, <5 %; moderate, 5–9.9 %; high, 10–14.9 %; and very high, ≥ 15 % [15]. This score is only applicable to individuals aged 35 to 74 years.

Modifiable major CVRFs registered in the EHR and the sum of these major factors were analysed: smoking, hypertension, hypercholesterolaemia and diabetes. Other CVRFs evaluated were: hypertriglyceridaemia, obesity, and alcoholism (in average units of weekly consumption, classified as: low risk consumption [17–28 units in men and 11–17 units in women]; risky consumption [>28 and > 17, respectively]) [16, 17]. Five additional variables were considered in the analysis: sex (female/male), age (years), age groups (19–24, 25–44, 45–64, 65–79, and 80+ years), number of chronic diseases and setting (urban/rural). Physical examinations yielded values for body mass index (BMI, kg/cm^2^) and blood pressure (mm Hg) and included laboratory tests: glycated haemoglobin (%), creatinine (mg/dl), uric acid (mg/dl), total cholesterol (mg/dl) and triglycerides (mg/dl), along with glomerular filtration rate < 60 ml/min/1.73 m^2^ to determine a decrease in renal function [18].

### Statistical analysis

Descriptive statistics were used to summarize overall information. Categorical variables were expressed as frequencies (percentage) and continuous as mean (Standard deviation, SD) or median (interquartile range, IQR). The MM crude prevalence and 95 % confidence intervals (CI) were calculated. The differences between MM and non-MM groups were tested using Student t, Mann–Whitney or Chi-square for unadjusted comparison, as appropriate. The crude prevalence (95 % CI) of MM-CMG was calculated. Prevalence estimates of each CV chronic condition and 95 % CI were obtained. The 95 % CI for the prevalences was calculated using the continuity-corrected Wilson score interval.

For comparison of MM-CMG, MM-NCMG and non-MM groups, ANOVA, Kruskal-Wallis or Chi square tests were used as appropriate. To determine the most prevalent MM patterns in MM-CMG, all possible combinations of each CV disease, one and two chronic conditions and their frequencies were calculated for each sex and age group. CVR score (REGICOR) distribution was compared within the three groups studied..We assumed that missing data were missing completely at random (MCAR), and so we performed a complete case analysis to handle missing data. We had sufficient power for our analysis, even though we lost part of our data set.

Spearman correlation between number of chronic diseases and chronic cardiovascular diseases and age was assessed.

All statistical tests were two-sided at the 5 % significance level. The analyses were performed using SPSS for Windows, version 18 and R version 3.2.3.

## Results

**Fig. 1:**
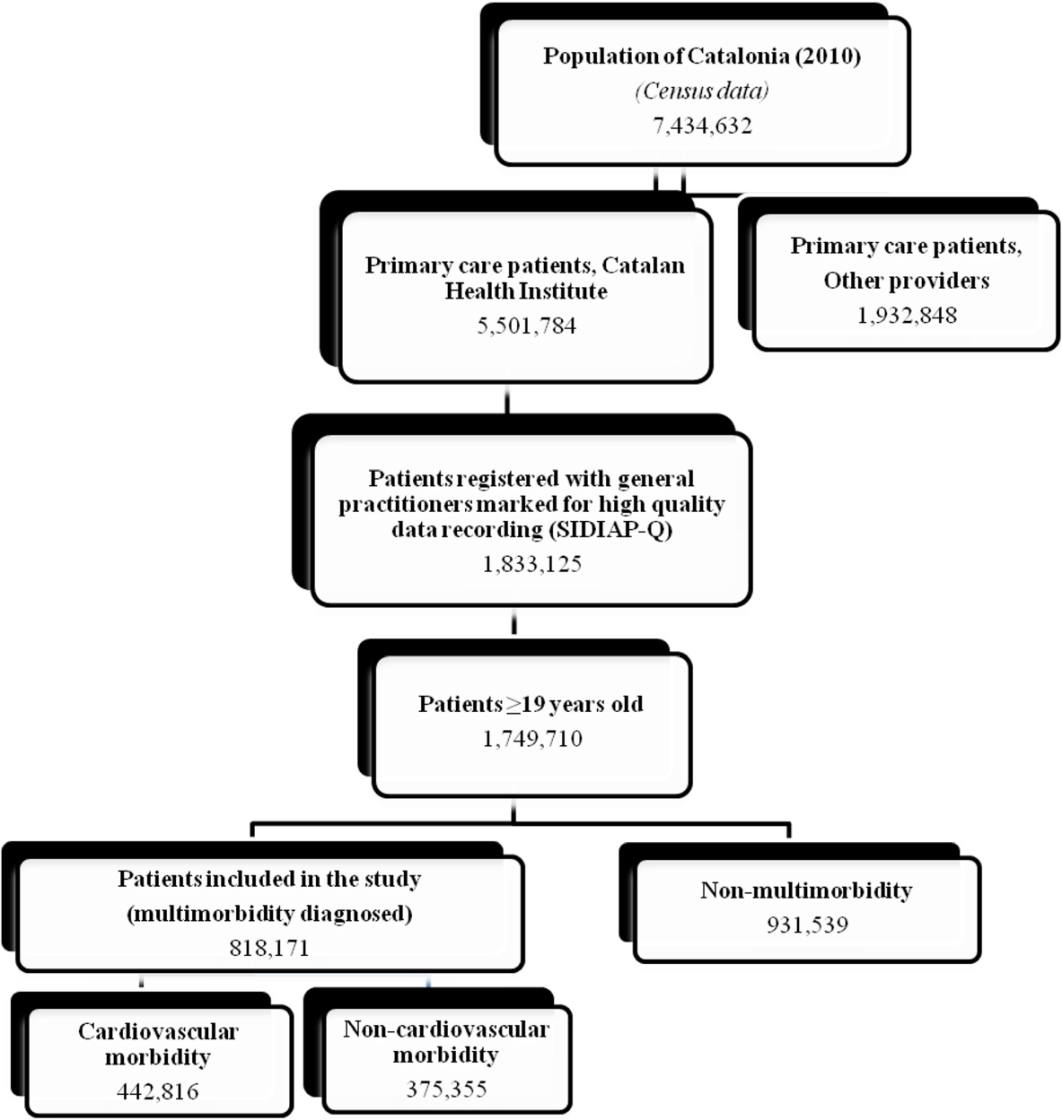
Sampling framework

A total of 1,749,710 individuals ≥19 years old were included in the study (Fig. 1). The mean age of the sample was 47.4 years (SD: 17.8) and 50.7 % were women. Nearly half of the total cohort (46.8 %, 95 % CI: 46.7–48.1) met MM criteria. Descriptive characteristics of patients in the MM group (including both the MM-CMG and MM-NCMG) and the non-MM group are shown in Table 1. For all variables, significant differences were found between the three study groups (Table 1).

**Table 1:** Descriptive characteristics of patients by multimorbidity or non-multimorbidity group (1,749,710 adult patients, Catalonia, 2010)

	Multimorbidity group		Non-multimorbidity group	Total
	Cardiovascular morbidity	Non-cardiovascular morbidity		
Variables	(*n* = 442,816)	(*n* = 375,355)	(*n* = 931,539)	(*N* = 1,749,710)
Age (years)	65.7 (14.6)	48.2 (15.4)	38.3 (12.7)	47.4 (17.8)
Sex (% females)	56.1	57.5	45.5	50.7
Number of chronic diseases (median, IQR)	5.0 (3.0;7.0)	3.0 (2.0;4.0)	0.0 (0.0;1.0)	1.0 (0.0;3.0)
Visits (median, IQR)	10.0 (6.0;18.0)	6.0 (3.0;11.0)	1.0 (0.0;4.0)	4.0 (1.0;9.0)
Urban setting (%)	83.4	84.0	84.2	84.0
Physical examination				
BMI (kg/cm^2^) (*n*= 509,368)	29.7 (5.1)	28.0 (5.4)	26.0 (4.7)	28.3 (5.3)
Systolic BP (mm Hg) (*n* = 773,446)	135.2 (16.2)	126.6 (14.6)	123.8 (14.9)	129.7 (16.3)
Diastolic BP (mm Hg) (*n* = 774,139)	76.7 (10.3)	76.0 (9.6)	74.5 (10.1)	75.9 (10.1)
Laboratory tests				
Glycated haemoglobin (%)(SD) (*n* = 208,581)	6.6 (1.3)	6.3 (1.5)	5.7 (1.2)	6.4 (1.4)
Creatinine (mg/dl) (SD) (*n* = 738,083)	0.9 (0.4)	0.8 (0.2)	0.8 (0.2)	0.9 (0.3)
Uric acid (mg/dl) (SD) (*n* = 475, 334)	5.5 (1.6)	4.8 (1.4)	4.8 (1.4)	5.2 (1.5)
Total cholesterol (mg/dl) (SD) (*n* = 745,010)	202.0 (39.7)	209.0 (40.6)	194.3 (38.4)	201.8 (40.0)
Triglycerides (mg/dl) (SD) (*n* = 190,159)	132.9 (82.9)	129 (98.7)	113.3 (75.7)	127.5 (86.4)
Glomerular filtration rate < 60 % (%) (*n* = 544,438)	17.6	3.2	1.3	9.2

*p*-values overall between multimorbidity and non-multimorbidity groups were significant for all variables (*p* < 0.001)

MM prevalence increased with age group (19–24, 25–44, 45–64, 65–79, and 80 or more years), as follows: 15.9 % (95 % CI: 15.7–16.1), 24.3 % (95 % CI: 24.2–24.4), 58.7 % (95 % CI:58.6–58.9), 88.7 % (95 % CI: 88.6–88.8), and 92.1 % (95 % CI: 91.9–92.2), respectively (*p* < 0.001).

The total MM population showed significantly higher prevalence (*p* < 0.001) than the non-MM group for three diagnsoses: hypertension (41.1 % vs. 1.8 %), hypercholesterolaemia (40.4 % vs. 3.2 %) and diabetes mellitus (15.6 % vs. 0.5 %). Except for smoking (23.1 % vs. 39.0 %, *p* < 0.001), the median number of CVRFs in the MM population was 4 (IQR: 2;6) vs. 2 (IQR: 1;4) in the non-MM group (*p* < 0.001). The median of major CVRFs in patients with MM was 1 (IQR: 1;2) and 0 (IQR: 0;1) for the non-MM group (*p* < 0.001) (Table 2).

**Table 2:** Distribution of cardiovascular risk factors by multimorbidity and non-multimorbidity groups (1,749,710 adult patients, Catalonia, 2010)

	Multimorbidity group		Non-multimorbidity group	Total
	Cardiovascular morbidity	Non-cardiovascular morbidity		
	(*n* = 442,816)	(*n* = 375,355)	(*n* = 931,539)	(*N* = 1,749,710)
Major modifiable cardiovascular risk factors				
Smoking (*n* = 1,329,331)	15.1	33.3	39.0	29.9
Hypertension (*n* = 1,749,710)	75.9	0.0	1.8	20.2
Hypercholesterolemia (*n* = 1,749,710)	47.7	31.8	3.2	20.6
Diabetes (*n* = 1,749,710)	22.8	7.1	0.5	7.6
Other factors				
Hypertriglyceridemia (*n* = 190,159)	27.8	24.9	18.2	24.9
Obesity (*n* = 1,749,710)	24.5	15.5	1.8	10.5
Alcoholism (*n* = 577,213)	28.2	29.8	32.4	29.9
Sum of major factors (median, [IQR]) (*n* = 1,329,331)	2 [1;2]	1 [0;1]	0 [0;1]	1 [0;1]
Cardiovascular risk score (REGICOR) (median, [IQR]) (*n* = 264,773)^a^	4 [3;7]	3 [2;5]	2 [1;4]	4 [2;6]
Cardiovascular risk score (REGICOR) (*n* = 264,773)^a^				
Low	60.6	74.6	84.5	67.4
Moderate	30.1	20.5	13.4	25.4
High	6.9	3.8	1.7	5.4
Very high	2.4	1.1	0.4	1.8

*p*-values overall between multimorbidity and non-multimorbidity groups were significant for all variables (*p* < 0.001)

^a^Only individuals aged 35 to 74 years were included because REGICOR score is only applicable to these age range

**Fig. 2:**
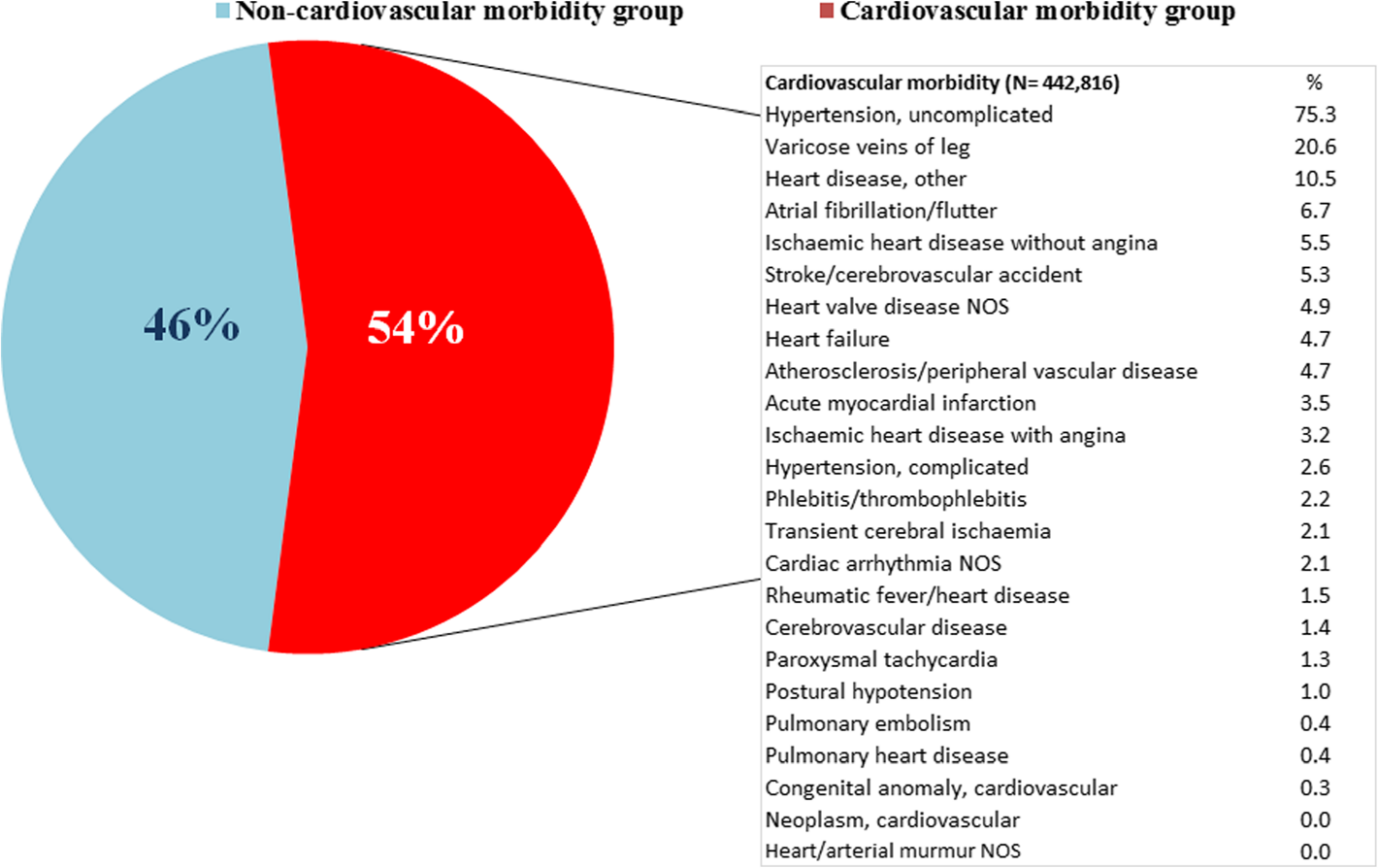
Cardiovascular morbidity burden in adults with multimorbidity (*n* = 818,171) in Catalonia, 2010

The MM-CMG patients constituted 54.1 % (95 % CI: 54.0–54.2) of the total MM population (Fig. 1). MM-CMG also increased with age group (19–24, 25–44, 45–64, 65–79, and 80 or more years), as follows: 1.6 % (95 % CI: 1.5–1.6), 5.0 % (95 % CI: 5.0–5.1), 29.4 % (95 % CI: 29.3–29.5), 68.9 % (95 % CI: 68.8–69.1), and 81.6 % (95 % CI: 81.6–81.4), respectively (*p* < 0.001). The four most prevalent cardiovascular diseases were uncomplicated hypertension (75.3 %), varicose veins of the leg (20.6 %), other heart disease (10.5 %), and atrial fibrillation/flutter (6.7 %) (Fig. 2). In this group, 38.2 % had more than one cardiovascular disease. Among those patients, the most prevalent combinations were the duet of uncomplicated hypertension & lipid disorder (38.8 %, 95 % CI: 38.7–39.0) and the triplet of uncomplicated hypertension & lipid disorder & non-insulin-dependent diabetes (11.3 %, 95 % CI: 11.2–11.4). By age group, the distribution of duets was uncomplicated hypertension & lipid disorder in all MM-CMG patients aged 45–80 years and also in younger men, aged 25–44 years; varicose veins of the leg & anxiety disorder/anxiety in women aged 19–44 years and uncomplicated hypertension & obesity in the youngest men, aged 19–24 (Table 3). The most prevalent triplets are shown in Table 4.

**Table 3:** Most prevalent comorbidities associated with cardiovascular diseases in multimorbid patients with cardiovascular morbidity, by age group and sex (442,816 adult patients, Catalonia, 2010)

Age Groups (years)	Female *n* = 248,407					Male *n* = 194,409				
	Cardiovascular index disease	Associated disease 1	*n*	%	95 % CI	Cardiovascular index disease	Associated disease 1	*n*	%	95 % CI
19–24 *n* = 2,152	Varicose veins of leg	Anxiety disorder/anxiety state	92	7.1	(5.8–8.7)	Hypertension, uncomplicated	Obesity	78	9.0	(7.2–11.2)
	Postural hypotension	Anxiety disorder/anxiety state	83	6.4	(5.2–8.0)	Heart disease, other	Acne	39	4.5	(3.3–6.2)
	Hypertension, uncomplicated	Obesity	35	2.7	(1.9–3.8)	Postural hypotension	Acne	16	1.8	(1.1–3.1)
	Paroxysmal tachycardia	Anxiety disorder/anxiety state	31	2.4	(1.7–3–4)	Varicose veins of leg	Acquired deformity of spine	16	1.8	(0.8–2.6)
	Heart disease, other	Anxiety disorder/anxiety state	24	1.9	(1.2–2.8)	Congenital anomaly cardiovascular	Acne	13	1.5	(0.8–2.6)
25–44 *n* = 37,308	Varicose veins of leg	Anxiety disorder/anxiety state	2,794	13.0	(12.6–13.5)	Hypertension, uncomplicated	Lipid disorder	3,425	21.6	(21.0–22.3)
	Hypertension, uncomplicated	Obesity	2,125	9.9	(9.5–10.3)	Varicose veins of leg	Lipid disorder	476	3.0	(2.7–3.3)
	Postural hypotension	Anxiety disorder/anxiety state	527	2.5	(2.3–2.7)	Heart disease, other	Lipid disorder	451	2.8	(2.6–3.1)
	Heart disease, other	Anxiety disorder/anxiety state	253	1.2	(1.0–1.3)	Hypertension, complicated	Hypertension, uncomplicated	201	1.3	(1.1–1.5)
	Paroxysmal tachycardia	Anxiety disorder/anxiety state	215	1.0	(0.9–1.1)	Acute myocardial infarction	Lipid disorder	159	1.0	(0.9–1.2)
45–64 *n* = 154,049	Hypertension, uncomplicated	Lipid disorder	25,930	32.5	(32.2–32.9)	Hypertension, uncomplicated	Lipid disorder	30,975	41.7	(41.3–42.0)
	Varicose veins of leg	Lipid disorder	10,339	13.0	(12.7–13.2)	Heart disease, other	Hypertension, uncomplicated	4,186	5.6	(5.5–5.8)
	Heart disease, other	Hypertension, uncomplicated	2,057	2.6	(2.5–2.7)	Ischaemic heart disease w/o angina	Lipid disorder	2,992	4.0	(3.9–4.2)
	Heart valve disease NOS	Hypertension, uncomplicated	1,131	1.4	(1.3–1.5)	Varicose veins of leg	Lipid disorder	2,745	3.7	(3.6–3.8)
	Stroke/cerebrovascular accident	Hypertension, uncomplicated	895	1.1	(1.1–1.2)	Acute myocardial infarction	Lipid disorder	2,582	3.5	(3.3–3.6)
65–79 *n* = 168,340	Hypertension, uncomplicated	Lipid disorder	46,236	49.5	(49.2–49.8)	Hypertension, uncomplicated	Lipid disorder	32,003	42.7	(42.3–43.1)
	Varicose veins of leg	Hypertension, uncomplicated	16,214	17.4	(17.1–17.6)	Heart disease, other	Hypertension, uncomplicated	8,156	10.9	(10.7–11.1)
	Heart disease, other	Hypertension, uncomplicated	7,075	7.6	(7.4–7.7)	Ischaemic heart disease w/o angina	Hypertension, uncomplicated	5,297	7.1	6.9–7.3)
	Atrial fibrillation/flutter	Hypertension, uncomplicated	4,739	5.1	(4.9–5.2)	Atrial fibrillation/flutter	Hypertension, uncomplicated	4,765	6.4	(6.2–6.5)
	Heart valve disease NOS	Hypertension, uncomplicated	4,164	4.5	(4.3–4.6)	Stroke/cerebrovascular accident	Hypertension, uncomplicated	4,522	6.0	(5.9–6.2)
80+ *n* = 80,967	Hypertension, uncomplicated	Lipid disorder	22,984	43.7	(43.3–44.2)	Hypertension, uncomplicated	Lipid disorder	9,115	32.1	(31.5–32.6)
	Varicose veins of leg	Hypertension, uncomplicated	8,923	17.0	(16.7–17.3)	Heart disease, other	Hypertension, uncomplicated	4,244	14.9	(14.5–15.4)
	Atrial fibrillation/flutter	Hypertension, uncomplicated	6,404	12.2	(11.9–12.5)	Atrial fibrillation/flutter	Hypertension, uncomplicated	3,600	12.7	(12.3–13.1)
	Heart disease, other	Hypertension, uncomplicated	6,022	11.5	(11.2–11.7)	Stroke/cerebrovascular accident	Hypertension, uncomplicated	2,651	9.3	(9.0–9.7)
	Heart failure	Hypertension, uncomplicated	5,814	11.1	(10.8–11.3)	Heart failure	Hypertension, uncomplicated	2,579	9.1	(8.7–9.4)

**Table 4:** Two most prevalent comorbidity diseases associated with cardiovascular diseases in multimorbid patients with cardiovascular morbidity, by age group and sex (442,816 adult patients, Catalonia, 2010)

Age Groups (years)	Women *n* = 248,407						Men *n* = 194,409					
	Index cardiovascular disease	Associated disease 1	Associated disease 2	n	%	95 % CI	Index cardiovascular disease	Associated disease 1	Associated disease 2	n	%	95 % CI
19–24 *n* = 2,152	Postural hypotension	Acne	Anxiety disorder/anxiety state	14	1.1	(0.6–1.9)	Hypertension, uncomplicated	Obesity	Acne	8	0.9	(0.4–1.9)
	Varicose veins of leg	Acne	Anxiety disorder/anxiety state	13	1.0	(0.6–1.8)	Heart disease, other	Dermatitis/atopic eczema	Asthma	6	0.7	(0.3–1.6)
	Hypertension, uncomplicated	Endocrine/metabolic/nutritional disorder, other	Obesity	9	0.7	(0.3–1.4)	Congenital anomaly cardiovascular	Asthma	Heart valve disease NOS	3	0.3	(0.1–1.1)
	Heart valve disease NOS	Acne	Acne	5	0.4	(0.1–1.0)	Cardiac arrhythmia NOS	Acne	Musculoskeletal disease, other	3	0.3	(0.1–1.1)
	Paroxysmal tachycardia	Depressive disorder	Anxiety disorder/anxiety state	4	0.3	(0.1–0.9)	Heart valve disease NOS	Asthma	Congenital anomaly, cardiovascular	3	0.3	(0.1–1.1)
25–44 *n* = 37,308	Hypertension, uncomplicated	Lipid disorder	Obesity	538	2.5	(2.3–2.7)	Hypertension, uncomplicated	Lipid disorder	Obesity	1,076	6.8	(6.4–7.2)
	Varicose veins of leg	Depressive disorder	Anxiety disorder/ anxiety state	491	2.3	(2.1–2.5)	Heart disease, other	Lipid disorder	Hypertension, uncomplicated	138	0.9	(0.7–1.0)
	Postural hypotension	Depressive disorder	Anxiety disorder/anxiety state	102	0.5	(0.4–0.6)	Varicose veins of leg	Lipid disorder	Obesity	105	0.7	(0.5–0.8)
	Phlebitis/thrombo-phlebitis	Obesity	Varicose veins of leg	59	0.3	(0.2–0.4)	Hypertension, complicated	Lipid disorder	Hypertension, uncomplicated	66	0.4	(0.3–0.5
	Heart disease, other	Depressive disorder	Anxiety disorder/anxiety state	46	0.2	(0.2–0–3)	Acute myocardial infarction	Lipid disorder	Hypertension, uncomplicated	60	0.4	(0.3–0.5
45–64 *n* = 154,049	Hypertension, uncomplicated	Lipid disorder	Obesity	9,134	11.5	(11.2–11.7)	Hypertension, uncomplicated	Lipid disorder	Diabetes, non-insulin dependent	8,631	11.6	(11.4–11.8)
	Varicose veins of leg	Lipid disorder	Hypertension, uncomplicated	3,761	4.7	(4.6–4.9)	Heart disease, other	Lipid disorder	Hypertension, uncomplicated	2,275	3.1	(2.9–3.2)
	Heart disease, other	Lipid disorder	Hypertension, uncomplicated	1,081	1.4	(1.3–1.4)	Ischaemic heart disease w/o angina	Lipid disorder	Hypertension, uncomplicated	1,744	2.3	(2.2–2.5)
	Heart valve disease NOS	Lipid disorder	Hypertension, uncomplicated	578	0.7	(0.7–0–8)	Acute myocardial infarction	Lipid disorder	Hypertension, uncomplicated	1,361	1.8	(1.7–1.9)
	Stroke/cerebrovascular accident	Lipid disorder	Hypertension, uncomplicated	500	0.6	(0.6–0.7)	Atherosclerosis/PVD	Lipid disorder	Hypertension, uncomplicated	1,275	1.7	(1.6–1.8)
65–79 *n* = 168,340	Hypertension, uncomplicated	Lipid disorder	Obesity	15,115	16.2	(15.9–16.4)	Hypertension, uncomplicated	Lipid disorder	Diabetes, non-insulin dependent	11,573	15.4	(15.2–15.7)
	Varicose veins of leg	Lipid disorder	Hypertension, uncomplicated	9,365	10.0	(9.8–10.2)	Heart disease, other	Lipid disorder	Hypertension, uncomplicated	4,389	5.9	(5.7–6.0)
	Heart disease, other	Lipid disorder	Hypertension, uncomplicated	4,298	4.6	(4.5–4.7)	Ischaemic heart disease w/o angina	Lipid disorder	Hypertension, uncomplicated	3,346	4.5	(4.3–4.6)
	Heart valve disease NOS	Lipid disorder	Hypertension, uncomplicated	2,530	2.7	(2.6–2.8)	Atherosclerosis/PVD	Lipid disorder	Hypertension, uncomplicated	2675	3.6	(3.4–3.7)
	Atrial fibrillation/flutter	Lipid disorder	Hypertension, uncomplicated	2,525	2.7	(2.6–2.8)	Stroke/cerebrovascular accident	Lipid disorder	Hypertension, uncomplicated	2,557	3.4	(3.3–3.5)
80+ *n* = 80,967	Hypertension, uncomplicated	Lipid disorder	Diabetes, non-insulin dependent	6,769	12.9	(12.6–13.2)	Hypertension, uncomplicated	Benign prostatic hypertrophy	Lipid disorder	3658	12.9	(12.5–13.3)
	Varicose veins of leg	Lipid disorder	Hypertension, uncomplicated	4,445	8.5	(8.2–8.7)	Heart disease, other	Lipid disorder	Hypertension, uncomplicated	1,823	6.4	(6.1–6.7)
	Heart disease, other	Lipid disorder	Hypertension, uncomplicated	3,255	6.2	(6.0–6.4)	Atrial fibrillation/flutter	Benign prostatic hypertrophy	Hypertension, uncomplicated	1,466	5.2	(4.9–5.4)
	Atrial fibrillation/flutter	Lipid disorder	Hypertension, uncomplicated	2,900	5.5	(5.3–5.7)	Ischaemic heart disease w/o angina	Lipid disorder	Hypertension, uncomplicated	1,357	4.8	(4.5–5.0)
	Heart failure	Lipid disorder	Hypertension, uncomplicated	2,776	5.3	(5.1–5.5)	Stroke/cerebrovascular accident	Lipid disorder	Hypertension, uncomplicated	1,153	4.1	(3.8–4.3)

**Fig. 3:**
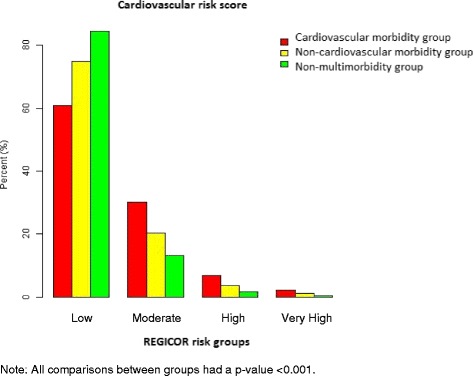
Cardiovascular risk distribution by multimorbidity or non-multimorbidity group (264,773 adult patients aged 35–74 years, Catalonia, 2010)

Significant differences were found in CVR scores between the MM-CMG, MM-NCMG and non-MM groups. Both MM groups had a higher CVR than the non-MM group (Fig. 3).

The correlations between number of chronic diseases and age was 0.60 (*p* < 0.001) and between number of chronic cardiovascular diseases and age was 0.57 (*p* 0.001).

## Discussion

### Main findings

Cardiovascular diseases accounted for more than 50 % of the burden of chronic disease in patients with MM patients, in part due to a high prevalence of hypertension. Both CVRFs and CVR were increased in patients with MM, compared to the non-MM population. Hypertension, diabetes and dyslipidaemia constituted the most prevalent cardiovascular-endocrine-metabolic pattern.

### Strengths and limitations

The major strength of this study was the analysis of a large, high-quality database of primary-care EHR that has been shown to be representative of a much larger population. Other studies have shown that more accurate conclusions can be drawn from EHR data than from survey-based datasets [19–21]. A number of potential limitations must be taken into account as well. First, chronic diseases could be underreported, which would affect the the observed MM prevalence. For example, 29 % of the Catalan population, especially people with higher socioeconomic status, buys private health insurance to supplement the universal health care services, which could affect population prevalence data. Other variables, such as smoking habit, are under-registered in the EHR (notably in the MM-CVG, exactly the group that should be addressed with precision on this variable), and this may modify the final results.

On the other hand, there could be an over-representation of chronic diagnoses (e.g., hypertension, diabetes, hyperlipidaemia, etc.) that are included in the goal/incentive contracts of Catalan PHCTs. Another aspect is the definition and counting of the chronic conditions included, which could modify the results as showen in previous studies [22, 23]. Finally, residual confounding cannot be completely excluded; certain epidemiological factors or other health determinants were not considered in this study, such as environmental factors.

### Comparison with existing literature

Very few studies have evaluated the cardiovascular burden in MM. In a sample of 73,254 persons of all ages, Landwehr et al. observed high comorbidity within a group of 30 cardiovascular disorders [24]. Another more recent study of cardiovascular diseases in patients with MM aged ≥18 years in a primary care setting showed that almost 60 % of the patients included had one cardiovascular disease and 34 % had two of them. Their study focused on ethnic differences and did not provide data about combinations of diseases or MM patterns by age groups [8]. Other studies have shown a high impact of cardiovascular diseases in middle-age, resulting in increased mortality risk [25, 26].

In addition, the prevalence of MM and the main patterns described in different studies focused in Spain may vary from other European countries. The number of chronic conditions included, age ranges of the study population and the definition of MM are the major explanations for these differences [9, 27]. Different Spanish regions also have differences in MM prevalences: 47 % (ages 19+) in Catalonia [28], 37 % (15+) in Aragon [9], and 24 % (all ages) in Basque Country [28], all from EHR analysis, and 69 % (18+) in a representative survey of the Spanish population [29]. These differences may be due to the methodology used in these studies or to the effects of socioeconomic inequalities between territorial regions of Spain [30]. Nonetheless, cardiovascular diseases are present in the main MM patterns reported in most countries. In a recent systematic review that included observational studies performed in primary care settings, hypertension and osteoarthritis were the most frequent combination, followed by other combinations that included cardiovascular diseases [1]. Similarly, in our study the most frequent combination was uncomplicated hypertension & lipid disorder.

Hypertension is the most prevalent disease related to medical consultation by those aged 65 and older [31]. The addition to hypertension of other cardiovascular diseases has been associated with increased morbidity and consequently with increased costs [32]. The heavy burden on health services indicates that cardiovascular morbidity requires major efforts in to prevent these diseases and especially a comprehensive approach to CVR prevention and reduction [33]. Although data about cardiovascular comorbidity are readily available, particularly with regard to a specific index disease (hypertension, coronary heart disease, heart failure, etc.) [34–36], published data on cardiovascular diseases in MM are scarce. In the primary health care setting, however, the MM approach has been recommended as more suitable than focusing on one specific disease [3, 37].

A recent literature review evaluated MM studies that specifically analysed cardiovascular diseases, and found that almost all were focused on an index disease (e.g., coronary heart disease or heart failure) or a limited number of cardiovascular diseases [38]. Another very recent study called for practice-based research focused on common dyads and triads in order to translate these results to best-practice guidelines, including disease patterns even when the relationships between them are not fully understood (for example, coexisting depression and CVD) [39]. Our study, evaluated the comorbidity of 24 chronic cardiovascular diseases, focusing the attention on these diseases and providing data on the patterns observed.

### Impact of the study

The findings from this study make several contributions to the current literature. Despite considerable knowledge about the separate impacts of cardiovascular diseases on the burden of chronic diseases, there is a gap in knowledge about their impact on MM as a whole. The present study quantifies this impact, based on analysis of a large, high-quality database. On the other hand, our data provide more information about the associations between cardiovascular diseases and MM, which may help clinicians to understand the complexity of MM. Our analysis of MM-CMG patterns showed a high prevalence of certain diseases accompanying cardiovascular diseases, such as lipid disorders in men aged 25 years and older and women aged 45 years and older. This could be a useful consideration for clinicians screening for comorbidities when a chronic disease is first diagnosed. Another finding of clinical interest was the association of depressive disorder and anxiety with paroxysmal tachycardia in younger women (19–24 years) and with heart disease in women aged 25–44 years. When the first of these cardiovascular diagnoses is made clinicians may be well advised to explore symptoms in the mental health sphere.

According to the observed presentation of associated diseases in the present study results, future studies could investigate the chronological order of diseases and the interventions in family practice that are most adequate to prevent the accumulation of diseases. Another potential impact of these results would be to introduce the concept of multimorbidity earlier in university degree programs that prepare health professionals, changing the disease-specific focus to a multimorbidity approach. Finally, in the field of health policy, efforts should focus on programs to avoid the accumulation of diseases when one disease appears, introducing guidelines on the management of associated diseases and the use of predictive algorithms that help doctors to identify occult diseases based on probabilistic models.

### Future research

Future research is needed to establish the optimal approaches to prevent or avoid the patient progression from non-MM to diagnsosis of one or more cardiovascular diseases in addition to other chronic but preventable diseases. In this sense, longitudinal studies should be performed to establish the specific moment of disease occurrence, how diseases are related over time and what interventions can be useful to prevent the onset of comorbidities in various patient populations.

## Conclusions

Data from a large sample of primary health care records showed that cardiovascular diseases represent more than half of the MM burden. Cardiovascular risk factors and risk profiles were higher in patients with MM than in the non-MM group, mainly due to the high prevalence of hypertension. Hypertension, diabetes and dyslipidaemia constitute the most prevalent cardiovascular-endocrine-metabolic pattern.

## Abbreviations

BMI: Body mass index
CVR: Cardiovascular risk
CVRF: Cardiovascular risk factors
EHR: Electronic health records
GP: General practitioners
ICD-10: International classification of diseases
ICPC: International classification of primary care
MM: Multimorbidity
MM-CMG: Multimorbidity- cardiovascular morbidity group
MM-NCMG: IDIAP Jordi Gol, Institut Universitari d’Investigació en Atenció Primària Jordi Gol
PHCT: Primary health care teams
REGICOR: *Registre Gironí del Cor*
SIDIAP: Information system for the development of research in primary care
